# Quinoidal Porphyrinoids as High-Performance Electron Acceptors for Organic Solar Cells: Design, Photophysics, and Device Optimization

**DOI:** 10.1021/acsami.6c06988

**Published:** 2026-07-02

**Authors:** Rubén Caballero, Fernando Langa, Rahul Singhal, Ganesh D. Sharma, Pilar de la Cruz

**Affiliations:** † Instituto de Nanociencia Nanotecnología y Materiales Moleculares (INAMOL), Universidad de Castilla-La Mancha, Toledo 45071, Spain; ‡ Department of Physics, 193160 Malviya National Institute of Technology, JLN Marg, Jaipur, Rajasthan 302017, India; § Department of Physics and Centre for Material Science and Nano-Electronics, The LNM Institute of Information Technology. Jamdoli, Jaipur, Rajasthan 302031, India

**Keywords:** quinoidal, porphyrinoid, porphyrins, nonfullerene acceptors, binary organic solar cells

## Abstract

Quinoidal porphyrinoids represent a promising class of electron accepting materials due to their extended π conjugation, strong electron affinity, and structural rigidity, yet their application in organic solar cells (OSCs) remains unexplored. This work presents the design and synthesis of a nickel based quinoidal porphyrinoid (**NiQP**) and its implementation as an n type acceptor in bulk heterojunction OSCs using PM6 as the donor polymer. **NiQP** exhibits broad absorption extending into the near-infrared region and a narrow optical bandgap of approximately 1.44 eV, enabling complementary light harvesting with PM6. PM6:**NiQP** devices deliver a power conversion efficiency (PCE) of 8.47% in as cast films, which increases to 12.51% after solvent vapor annealing (SVA), mainly due to enhanced short circuit current density and fill factor. Photophysical and electrical analyses show that SVA improves nanoscale morphology, exciton diffusion, and dissociation efficiency, while suppressing bimolecular and trap assisted recombination. Energy loss analysis further indicates reduced radiative and non radiative recombination losses in SVA treated devices, accompanied by lower Urbach energy and diminished energetic disorder. These results demonstrate the potential of quinoidal porphyrinoids as efficient electron acceptors and provide guidelines for molecular design and processing strategies in next generation OSCs.

## Graphical abstract

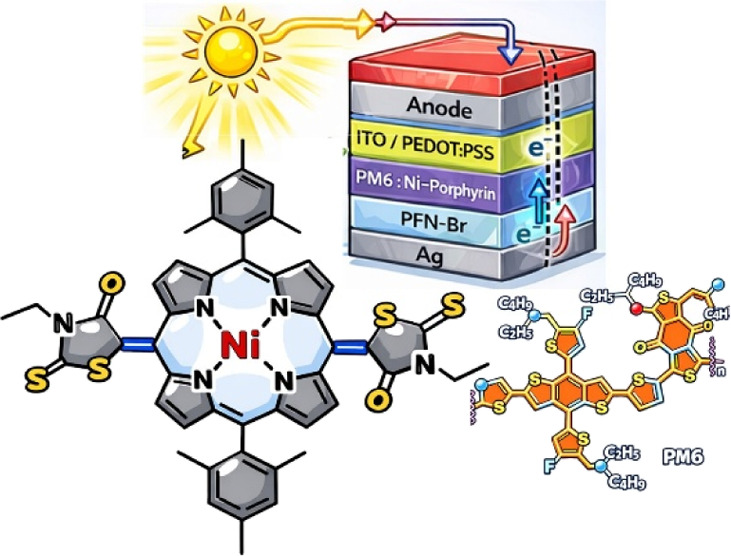

## 1 Introduction

The development of efficient n-type organic semiconductors is critical for advancing optoelectronic technologies, including organic field-effect transistors (OFETs),^1^ organic light-emitting diodes (OLEDs),,,^2–4^ organic thermoelectrics (OTEs),^5^ and organic photovoltaics (OPVs).^6–8^ Among these, high-performance OPV materials represent a major research focus, with power conversion efficiencies (PCEs) now exceeding 20%.^9,10^

Despite these advances, n-type semiconductors generally exhibit inferior performance compared to their p-type counterparts. This disparity primarily arises from the difficulty of designing molecules with a lowest unoccupied molecular orbital (LUMO) energy level below −4.0 eV while maintaining stability under ambient conditions.^11^ Quinoidal compounds have emerged as highly promising molecular platforms to address this challenge,^5,12^ owing to their distinctive structural and electronic attributes that make them particularly suitable for next-generation organic electronic applications: (a) Planar and rigid architecture, which promotes efficient π–π stacking in the solid state. This structural feature facilitates close molecular packing and enhances intermolecular charge transport. (b) Extended conjugation and strong π-electron delocalization, leading to reduced optical bandgaps and broadened absorption profiles. These properties enable effective light harvesting across visible and near-infrared regions. (c) Intrinsic electron-deficient character, which induces pronounced intramolecular charge-transfer interactions, stabilizes radical states, and improves electronic conductivity. d) High electron affinity, making quinoidal derivatives excellent candidates for n-type semiconductors and electron-accepting materials. Their electronic properties can be finely tuned through structural modifications, allowing precise control over HOMO–LUMO energy levels having exceptional chemical and thermal stability, ensuring durability and operational reliability in optoelectronic devices. Collectively, these characteristics position quinoidal compounds as versatile and robust building blocks for the design of high-performance n-type semiconductors in organic electronics^13^ and particularly, OSCs.^6,14^

Porphyrins are π-conjugated macrocycles exhibiting outstanding optical and electronic characteristics, which have attracted significant attention in OSCs. Their well-established synthetic versatility facilitates the design of diverse molecular architectures through relatively few synthetic transformations.^15^ In this sense, by chemical derivatization in meta- and beta-positions or changing the central metal, their frontier orbitals can be tailored to be used as p-type or n-type components in OSCs.^16^

Although porphyrins are traditionally regarded as electron-rich π-conjugated systems and are widely employed as electron-donating units, their electronic properties can be significantly modulated through rational structural modification. In particular, the incorporation of electron-withdrawing fragments, quinoidal contributions, and extended π-conjugation may substantially stabilize the LUMO energy level and induce pronounced electron-accepting character. In the present system, these electronic effects alter the conventional donor nature of the porphyrinic framework and promote charge-accepting behavior. Moreover, porphyrinic acceptors retain several advantageous features, including intense and broad visible-light absorption, efficient π-electron delocalization, and high structural tunability, which may be beneficial for charge-transfer processes and optoelectronic applications.

Although scarcely studied, quinoidal porphyrinoids^17–20^ have been shown to exhibit different electronic properties, including shifted absorption to the infrared region and enhanced two-photon absorption, compared with aromatic porphyrins.^21^ However, its use in OSCs as an n-type component remains unexplored. In this work, we have designed a new Ni porphyrinoid-based acceptor denoted as **NiQP** (Chart 1), which exhibits broad absorption spectra covering the visible-to-NIR region of the solar spectrum and a narrow optical band of 1.52 eV. For the construction of the devices, we have used a well-known polymer, PM6, as a donor that exhibits a complementary absorption spectrum with **NiQP**. The BHJ OSCs based on as-cast and solvent vapor annealed PM6:**NiQP** active layer showed 8.47% and 12.51%, respectively.

**Chart 1:**
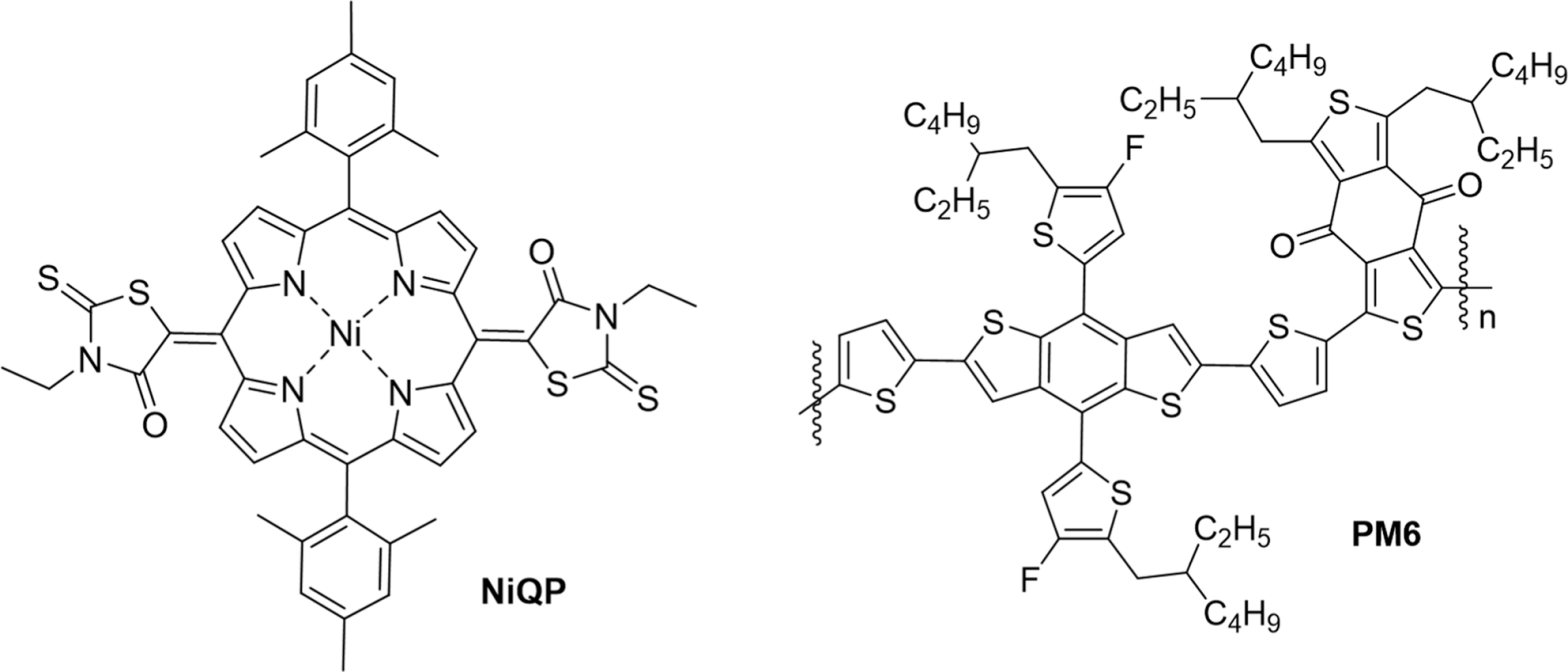
Chemical structures of **NiQP** and PM6.

## 2 Results and Discussion

### 2.1 Synthesis and Optical and Electrochemical Properties

The target compound **NiQP** was isolated as a dark green solid in 83% yield from Ni-porphyrin **1** ^22^ and rhodanine (**2**) followed by oxidation with DDQ (Scheme 1). **NiQP** is obtained as an approximately 1:1 mixture of cis/trans isomers and fully characterized by means of ^1^H NMR, ^13^C NMR and ESI-QTOF (Figures S1–S4). Consistent with the literature,^19,23^ the loss of aromaticity leading to a quinoidal porphyrinoid, is evidenced by the upfield shift of the β pyrrolic protons to 6–7 ppm, instead of the typical ∼9 ppm observed for aromatic porphyrins in the ^1^H NMR spectrum. The loss of planarity, also reported for other quinoidal porphyrinoids, is further supported by the splitting of the ^1^H and ^13^C NMR signals corresponding to the methyl groups.

**Scheme 1:**
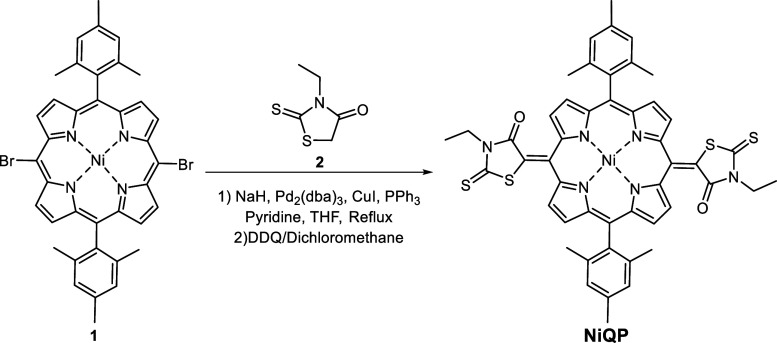
Synthetic Route to **NiQP**

The absorption spectra of **NiQP** in dilute chloroform solution and as a thin film are presented in Figure 1a. In solution, the spectrum displays broad and intense Soret and Q bands, with maxima at 462 nm (log ε = 4.77) and a wide absorption region from 600 to 900 nm, featuring a peak at 725 nm with a high molar extinction coefficient (log ε = 4.57). Compared to the diluted solution, the Q-band in the thin film exhibits a pronounced red shift (38 nm relative to the solution) and enhanced intensity, which can be attributed to *J*–*J* aggregation in the solid state. The optical bandgap, estimated from the onset of the thin-film absorption, is approximately 1.44 eV. The donor polymer PM6 absorbs in the 500–650 nm range, where **NiQP** shows minimal absorption, indicating excellent spectral complementarity. Figure S6 shows the absorption spectra of the optimized as-cast and SVA-treated PM6:**NiQP** blend films. The characteristic absorption bands of both **NiQP** and PM6 are clearly visible in both films. Notably, the SVA-treated film exhibits a slight red shift relative to the as-cast film, which may be mainly attributed to SVA-induced recrystallization and molecular rearrangement. These processes can modify the film morphology and optoelectronic properties by enhancing intermolecular interactions and promoting more ordered molecular packing.^24^

**Figure 1:**
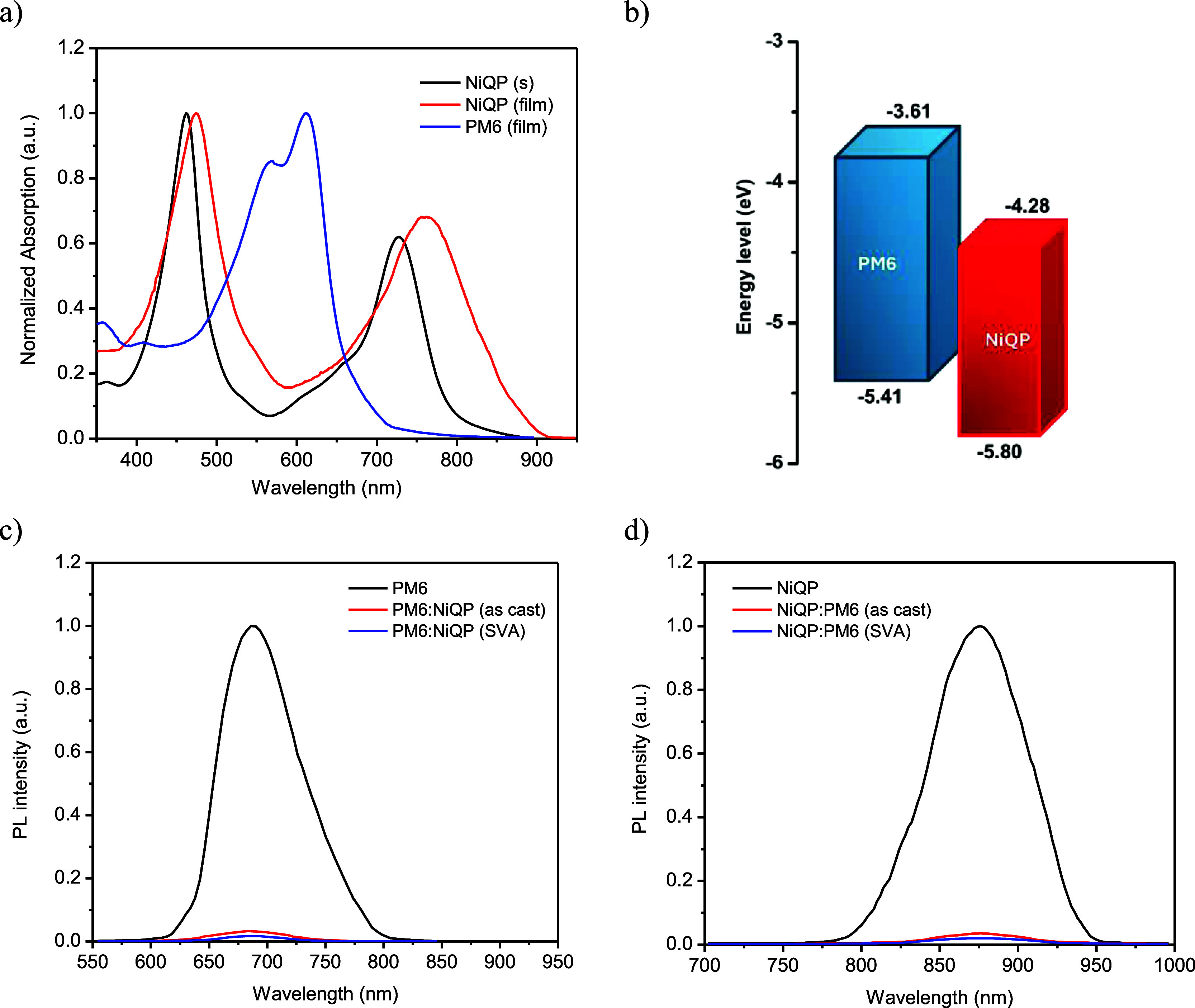
(a) Optical absorption spectra of the **NiQP** (sol), **NiQP** (film) and PM6 (film); (b) Energy levels diagram of the **NiQP** and PM6 and Photoluminescence (PL) spectra recorded for: (c) pristine PM6 and its blend with **NiQP** (excited at 610 nm) and (d) pristine **NiQP** and its blend with PM6 (excited at 750 nm).

The frontier molecular orbital (FMO) energy levels of **NiQP** were investigated through the electrochemical Osteryoung square wave voltammetry (OSWC) and cyclic voltammetry (CV) (Figure S7). The HOMO and LUMO energy levels are estimated to be −5.80 and −4.28 eV, respectively. The deep HOMO energy level of **NiQP** is beneficial for the air stability. As compared to **NiQP**, donor polymer PM6 showed HOMO and LUMO levels of −5.41 and 3.61 eV, respectively, with HOMO and LUMO energy offsets between **NiQP** and PM6 are suitable for electron transfer from the LUMO of PM6 to the LUMO of **NiQP** and hole transfer from HOMO of the **NiQP** to the HOMO of PM6, after the generation of excitons and dissociation in the active layer.

### 2.2 Computational Studies

The optimized geometry obtained at the DFT-B3LYP/6-31G/LANL2DZ level reveals that the Ni-porphyrinoid derivative exhibits pronounced out-of-plane (OOP) distortions accompanied by substantial meso-aryl twisting. As shown in Figure S8a, the macrocycle adopts a bent conformation in the side view (50.4°) together with a near-orthogonal dihedral in the top view (86.8°). This nonplanar arrangement is consistent with the ruffling/saddling balance reported for quinoidal porphyrinoids^23^ and meso-phenyl nickel porphyrins, in which the porphyrin core remains approximately square-planar around the Ni center while the meso-aryl substituents rotate significantly due to steric congestion.^25–27^

The electrostatic potential (ESP) surface (Figure S8b) displays a well-defined push–pull distribution characterized by electron-rich regions on donor fragments and electron-deficient zones on acceptor domains. The resulting electronic anisotropy supports efficient intramolecular charge transfer (ICT) and explains the large dipole moment (μ ≈ 6.0 D) derived from this analysis (Figure S6b).

The frontier molecular orbitals have also been determined by theoretical calculations (Figure S9) finding these values: E­(_HOMO_) = −5.75 eV, E­(_LUMO_) = −3.77 eV, and an energy gap Δ*E* = 1.98 eV. The HOMO isosurface shows extensive delocalization over the conjugated macrocycle with notable electron density around the Ni center, while the LUMO remains localized on the porphyrinoid core, extending primarily over the β-carbons and methine bridges, and involving the meso-aryl units only marginally. This spatial distribution is, also, in full agreement with prior DFT studies of Ni-diphenylporphyrins, which consistently report HOMO localization on the Ni–macrocycle framework and LUMO confinement to the inner conjugated ring, resulting in an efficient ICT pathway across the macrocycle.

The electronic structure of **NiQP**, featuring a low-lying LUMO (−3.77 eV) and strong push–pull character, makes it a suitable electron acceptor. Its energy alignment with PM6 enables efficient charge transfer at the donor–acceptor interface.

### 2.3 Photoluminescence Spectra Analysis

Photoluminescence (PL) spectra were recorded for pristine PM6 (Figure 1c) and its blend with **NiQP** (excited at 610 nm), as well as for pristine **NiQP** (Figure 1d) and its blend with PM6 (excited at 750 nm). Upon excitation at 610 nm, pristine PM6 exhibited a strong PL emission peak at 692 nm, which was significantly quenched in the blend with **NiQP**. Similarly, when excited at 750 nm, pristine **NiQP** showed intense PL peak at 876 nm. In the blend with PM6, the PL peak at 876 nm was strongly quenched, and this quenching was further enhanced after solvent vapor annealing (SVA). PL quenching upon excitation at 610 nm is attributed to electron transfer from PM6 to **NiQP**, whereas quenching upon excitation at 750 nm is ascribed to hole transfer from **NiQP** to PM6. Overall, PL quenching is more pronounced in the SVA-treated blend across all excitation wavelengths, indicating more efficient charge transfer in the bulk heterojunction (BHJ) blend than in the as-cast film, likely due to a more favorable nanoscale morphology. It can be seen from Figure S10a that pristine **NiQP** film showed a strong PL peak at 576 nm, but when excited at 450 nm, but when blended with PM6, PL remains similar, i.e., no PL quenching. However, the PL spectrum of **NiQP** excited at 450 nm strongly overlaps with the absorption spectrum of PM6 (Figure S10b), implying that excitons generated in **NiQP** are transferred to PM6 and subsequently dissociated into free carriers. We infer that excitons generated in **NiQP** upon absorption of photons near 460 nm are effectively utilized through energy transfer to PM6.

### 2.4 Photovoltaic Properties

The photovoltaic performance of the blend PM6:**NiQP** was evaluated using the BHJ active layer composed of PM6 and **NiQP** within a conventional device structure: ITO/PEDOT/PSS/active layer/PFN-Br/Ag. PM6 was selected as the polymer donor since it exhibits absorption profile complementary to that of **NiQP**, enabling panchromatic absorption in the PM6:**NiQP** active layer, which is advantageous for the light-harvesting efficiency in OSCs. Initially, devices were fabricated with varying PM6:NiQP weight ratios using chloroform as the processing solvent, and the optimal performance was achieved for PM6:**NiQP** (1:1.2). The photovoltaic parameters for OSCs based PM6:NiQP for different weight ratios between PM6 and NiQP are compiled in Table S1. Subsequently, the PM6:**NIQP** (1:1.2) active layer was subjected to SVA by exposure to THF vapors for 40 s. Figure 2a shows the current–voltage (J–V) characteristics of as-cast and SVA-treated OSCs under AM1.5 G (100 mW/cm^2^), and the corresponding photovoltaic parameters are summarized in Table 1. The as-cast OSCs showed an overall PCE of 8.47% (*J*_SC_ = 16.45 mA/cm^2^, *V*_OC_ = 0.883 V, and FF = 0.583), which increased after SVA treatment to 12.51% (*J*_SC_ = 21.76 mA/cm^2^, *V*_OC_ = 0.854 V, and FF = 0.673), demonstrating the effectiveness of SVA treatment in the blend. The slight reduction in *V*_OC_ observed for the SVA-treated OSC may be associated with increased radiative recombination. Nevertheless, despite this minor decrease in *V*_OC_, the SVA-treated device exhibited more efficient exciton dissociation, leading to an enhanced *J*_sc_.^28^ The external quantum efficiency (EQE) spectra of the OSCs based on the as-cast and SVA-treated active layer are shown in Figure 2b. The PM6:**NiQP**-based OSCs exhibit a broadband photoresponse from 350 to 900 nm, closely matching the absorption spectra of the as-cast and SVA-treated blend films, indicating that photon absorption by both PM6 and **NiQP** contributes to photocurrent generation. The as-cast device showed a maximum EQE of ∼55.9%, which increased to ∼69.7% after SVA treatment. The *J*_SC_ values calculated from EQE spectra were 16.18 and 21.45 mA cm^–2^ for as-cast and SVA-treated devices, respectively, in good agreement with those obtained from J–V measurements under illumination.

**Figure 2:**
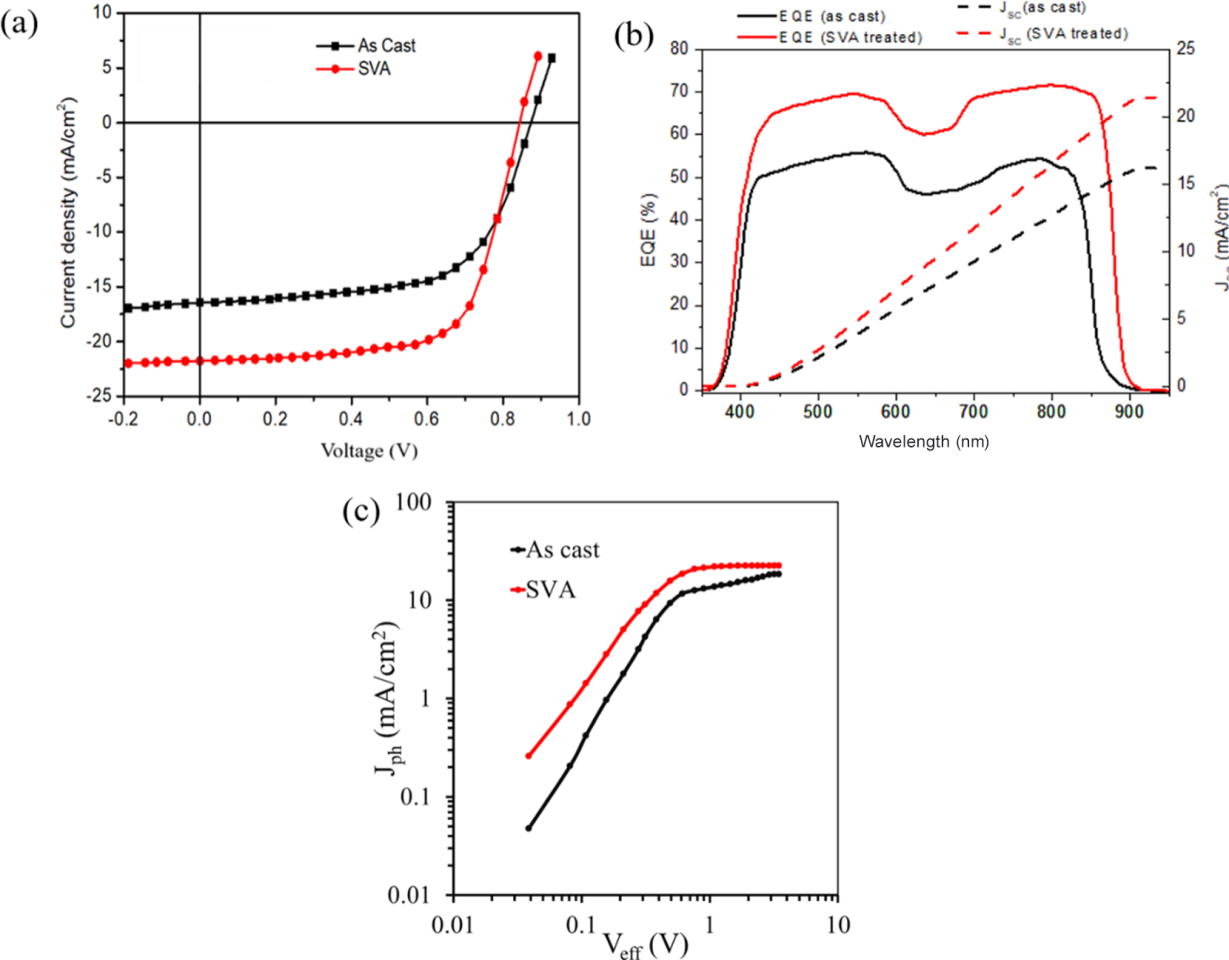
(a) (a) J–V characteristics under 1 sun condition (AM1.5G, 100 mW cm^–2^); (b) EQE spectra, and (c) Variation of *J*_ph_ with *V*_eff_ for optimized binary PM6:**NiQP**-based OSCs (as cast and SVA).

**Table 1:** Photovoltaic Parameters of the Binary OSCs Based on PM6 and NiQ**P**

Active layer	*J*_SC_ (mA/cm^2^)	*V*_OC_ (V)	FF	PCE (%)
PM6:**NiQP** (as cast)	16.45 (16.23 ± 0.15)	0.883 (0.878 ± 0.004)	0.583 (0.578 ± 0.003)	8.47 (8.26 ± 0.25)^a^
PM6: **NiQP** (SVA)	21.76 (21.48 ± 0.18)	0.854 (0.846 ± 0.005)	0.673 (0.668 ± 0.004)	12.51 (12.24 ± 0.23)^a^

a Average of 8 devices.

### 2.5 Exciton Generation, Dissociation, and Charge Transport Analysis

The photovoltaic performance of OSCs based on the SVA-treated PM6:**NiQP** active layer was significantly enhanced, primarily due to improved *J*_SC_ and FF values. To elucidate the origin of these improvements, we analyzed exciton generation and diffusion toward donor/acceptor interfaces, followed by dissociation and charge transport. The relationship between photocurrent density (*J*_ph_) and effective voltage (*V*_eff_)^29^ is shown in Figure 2c, with details of *J*_ph_ and *V*_eff_ estimation provided in the Supporting Information. As illustrated, *J*_ph_ initially increases with *V*_eff_ and then approaches a saturation value (*J*_sat_) at high *V*_eff_. For the SVA-treated OSC, *J*_ph_ saturates at approximately 1.18 V, indicating that most photogenerated excitons reaching the D/A interfaces successfully dissociate into free carriers. However, complete saturation is not achieved even at the highest *V*_eff_, likely due to field-dependent charge generation and space-charge effects^30,31^ The maximum exciton generation rate (*G*_max_) was calculated as *G*_max_ = *J*_sat_/(qL), where q is the elementary charge and L is the active layer thickness. *G*_max_ values were approximately 1.12 × 10^28^ and 1.36 × 10^28^ m^–3^ s^–1^ for OSCs based on as-cast and SVA-treated active layers, respectively, consistent with the higher EQE observed for the latter. Exciton dissociation probability (P_diss_) and charge collection probability (P_coll_) were estimated from the ratios *J*_sat_/*J*_ph_ under short-circuit conditions and at the maximum power point, respectively. P_diss_/P_coll_ values were 0.867/0.698 and 0.963/0.754 for as-cast and SVA-treated devices, respectively, confirming that SVA treatment enhances both exciton dissociation and charge collection.

Exciton diffusion toward donor–acceptor (D/A) interfaces and subsequent dissociation were further investigated using time-resolved photoluminescence (TRPL) measurements of pristine PM6 (as-cast and SVA-treated) and their blends with **NiQP**, excited at 610 nm and probed at 692 nm, as shown in Figure 3a,b, respectively. The PL decay time in pristine PM6 provides the exciton lifetime (τ_ex_), which in turn determines the exciton diffusion length (L_D_) according to L_D_ = (D τ_ex_)^1^´^2^. The τ_ex_ value extracted from monoexponential fitting of TRPL data for the SVA-treated PM6 film is approximately 0.827 ns, higher than that of as-cast PM6 (0.595 ns). The longer τ_ex_ for SVA-treated PM6 results in a greater L_D_, indicating that more excitons can reach D/A interfaces before recombination in SVA-treated OSCs compared to as-cast devices.

**Figure 3:**
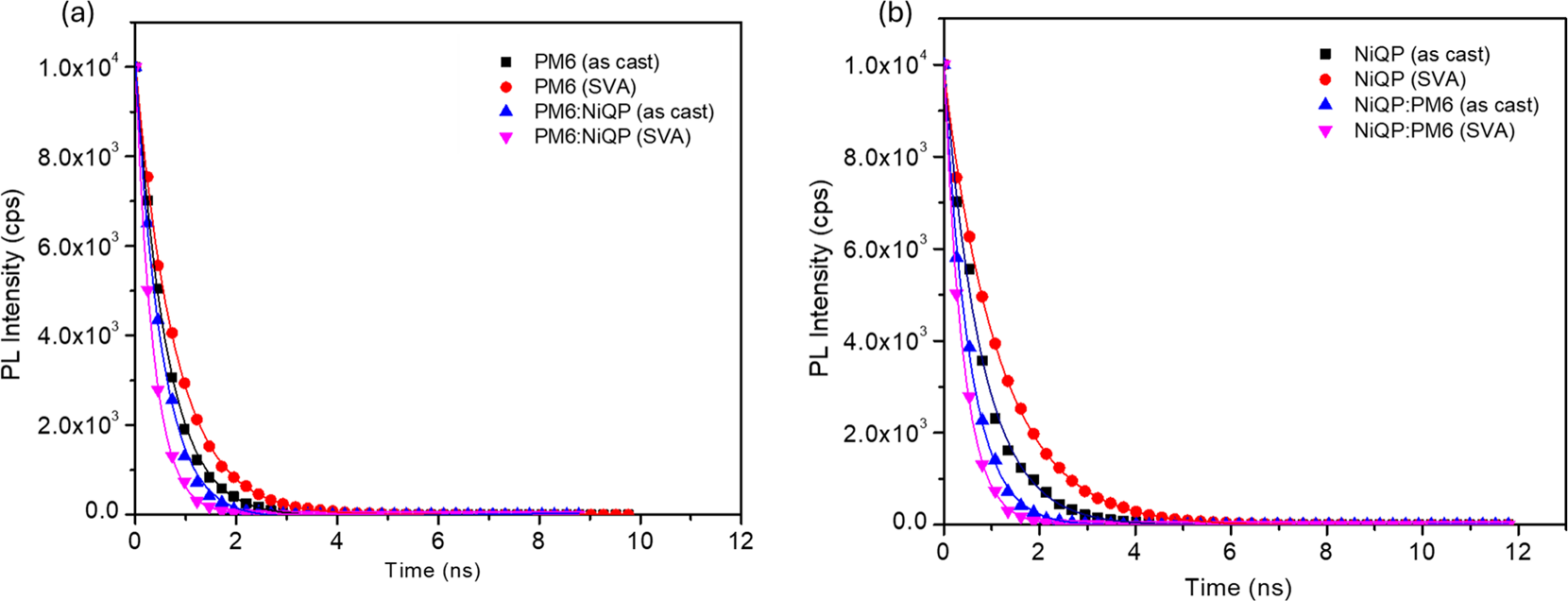
TRPL plots for (a) pristine PM6 and PM6:**NiQP** films and for (b) pristine **NiQP** and PM6:**NiQP** films.

PL decay in blended films reflects the degree of exciton dissociation. The exciton dissociation time (τ_exdiss_) for SVA-treated PM6:**NiQP** is about 0.335 ns, shorter than that of the as-cast blend (0.464 ns). The exciton dissociation efficiency (η_exdiss_) was calculated using η_exdiss_ = (1/τ_exdiss_)/[(1/τ_exdiss_) + (1/τ_ex_)].^32,33^ The η_exdiss_ values are 56.12% and 71.18% for OSCs based on as-cast and SVA-treated active layers, respectively.

Similarly, η_exdiss_ for excitons generated in the **NiQP** phase was estimated via TRPL measurements of pristine **NiQP** and its blends (as-cast and SVA-treated), excited at 750 nm and probed at 876 nm (Figure 3b). The η_exdiss_ values for **NiQP** were 59.45% and 73.58% for as-cast and SVA-treated devices, respectively. These results demonstrate that SVA treatment significantly enhances exciton dissociation efficiency, consistent with *J*_ph_–*V*_eff_ analysis, leading to higher *J*_sc_ and FF and thus improved PCE. Furthermore, SVA treatment increases exciton lifetime and diffusion length, facilitating exciton migration to D/A interfaces before decay. In blended films, TRPL measurements reveal faster exciton quenching, indicating more efficient dissociation at D/A interfaces.

The dependence of *J*_SC_ and *V*_OC_ on illumination intensity (P_in_) is commonly used to gain insight into charge recombination processes in organic solar cells (OSCs)^34,35^ and is illustrated in the Figure 4a,b. The variation of *J*_SC_ with P_in_ follows the relationship: *J*_SC_ ∝ (P_in_)^α^, where α reflects the extent of bimolecular recombination (Figure 4a). For an ideal device without bimolecular recombination, α = 1. The calculated values of α for the as-cast and solvent vapor annealed (SVA)-treated devices are 0.918 and 0.966, respectively, indicating that SVA treatment of the active layer effectively suppresses bimolecular recombination.

**Figure 4:**
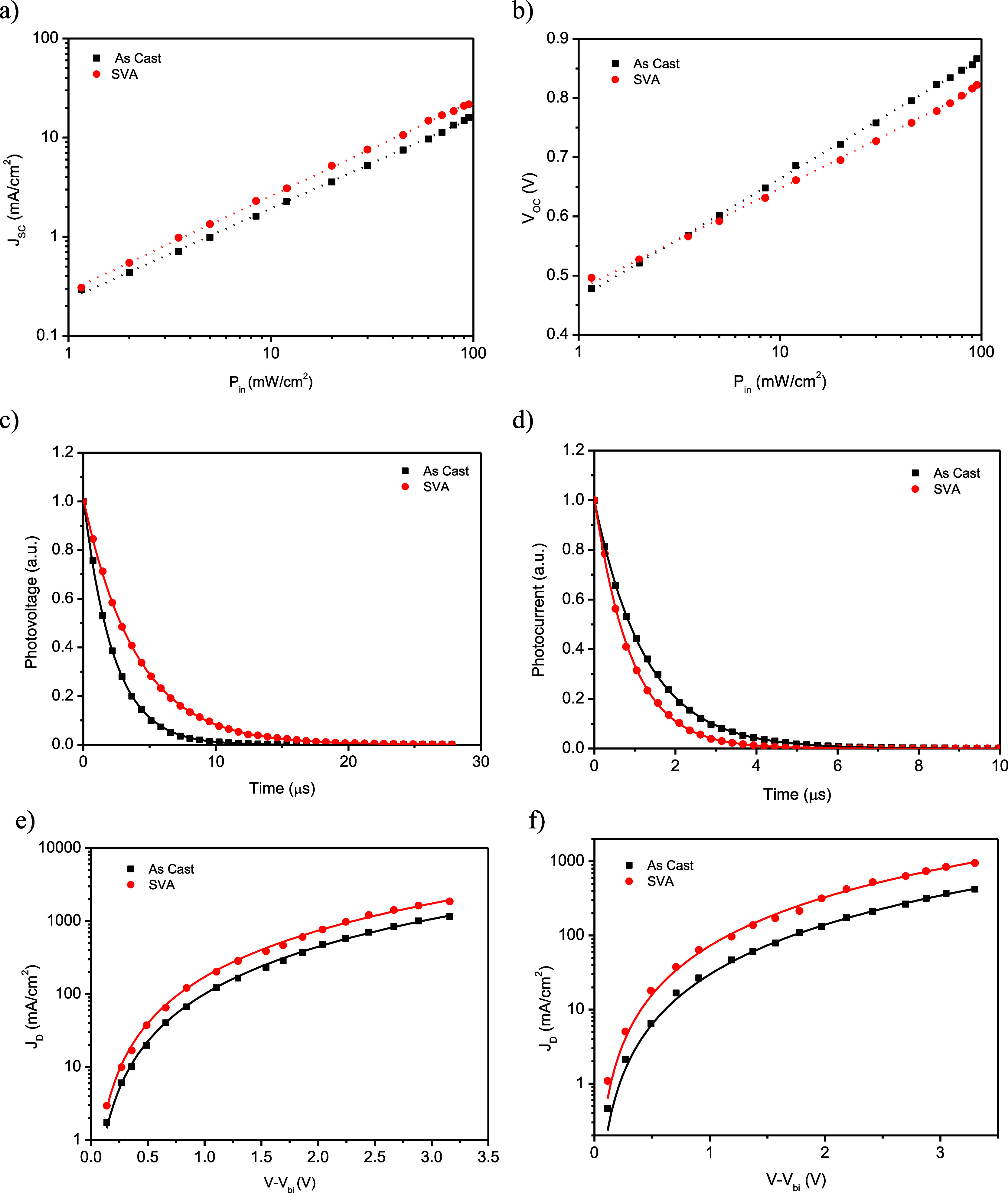
Variation of (a) *J*_SC_ and (b) *V*_OC_ with P_in_ and dark J–V characteristics for (c) TPV, (d) TPC (e) hole-only and (f) electron-only for binary **PM6**:**NiQP** devices, as cast and SVA.

Similarly, the variation of *V*_OC_ with *P*_in_ follows *V*_oc_= (*nk*T/q) ln­(P_in_) where n characterizes the degree of trap-assisted recombination (Figure 4b). The extracted *n* values for as-cast and SVA-treated PM6:**NiQP** devices are approximately 1.36 and 1.26, respectively, suggesting that SVA treatment reduces trap-assisted recombination. This improvement is attributed to the more favorable nanoscale morphology of the active layer achieved through SVA treatment.

Furthermore, we employed transient photocurrent (TPC) and transient photovoltage (TPV) measurements to conduct the depth of charge extraction dynamics and carrier lifetime (Figure 4c,d). TPV results (Figure 4c) show that the carrier lifetimes of as-cast and SVA-treated devices are 3.45 and 5.38 μs, respectively, indicating that the SVA treatment significantly suppresses the charge recombination processes. TPC results (Figure 4d) further reveal that the SVA treatment of the active layer shortens the charge extraction time to 0.978 μs, as compared to as-cast counterpart (1.14 μs), which fully demonstrates the significant enhancement effect of SVA treatment on the charge transport and extraction efficiency.

The hole and electron mobilities of the PM6:**NiQP** active layers were estimated by measuring the dark **J**–**V** characteristics of hole-only and electron-only devices, respectively, and fitting the data to a space-charge-limited current (SCLC) model^36^ (see Figure 4e,f). The extracted values of hole (Figure 4e) and electron mobilities (Figure 4f), along with their ratios, are presented in Figure 5a. Both electron and hole mobilities increased after treatment, with a low hole-to-electron mobility ratio of 1.29, indicating more balanced transport. This balance is beneficial for suppressing charge recombination, thereby enhancing *J*_SC_and the fill factor (FF) of the OSCs.

**Figure 5:**
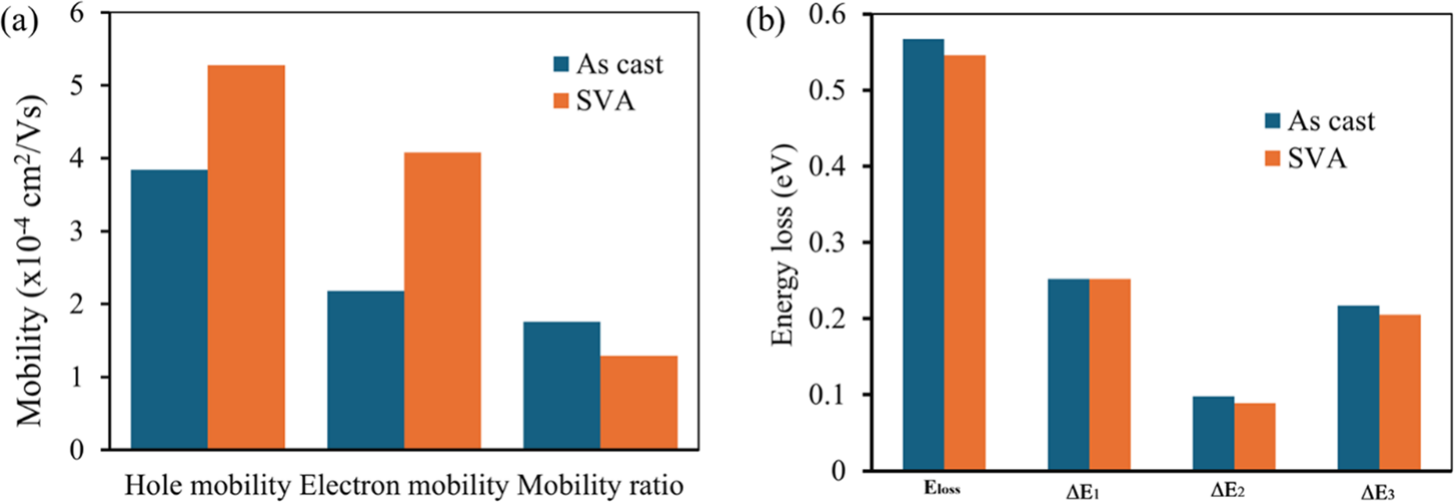
(a) Hole and electron mobilities and (b) Energy loss of as cast and SVA blends.

Since trap-assisted recombination is strongly influenced by the trap density (*N*_*t*_) in the active layer, *N*_*t*_ was estimated from the dark J–V characteristics using the SCLC model (see Figure S11). The calculated value of *N*_*t*_ is estimated as^37^ N_t_ = 2εε_o_V_TFL_/qL^2^ where V_TFL_ is the trap-filled limit voltage, ε is the dielectric constant of the active layer, ε_o_ is the permittivity of free space (8.854 × 10^–4^ F/cm), and L is the thickness of the active layer. The value of V_TFL_ is estimated from the intersection of ohmic and trap-free SCLC, which are 0.784 and 0.635 V, for the as-cast and SVA-treated devices, respectively. The calculated trap densities (N_t_) for the SVA-treated active layer (2.34 × 10^16^ cm^–3^) are lower than those for the as-cast active layer (2.76 × 10^16^ cm^–3^), further confirming the suppression of trap-assisted recombination in SVA-treated active layer.

### 2.6 Energy Loss Analysis

The energy loss (*E*_loss_) in these OSCs was systematically analyzed. The total *E*_loss_ was determined according to *E*_loss_ = *E*_g_ - *qV*_OC_, where *E*_g_ denotes the optical bandgap, estimated for the intersection point of the d­(EQE)/d*E* curve and the normalized EQE spectrum (Figure S12).^38,39^ The E_g_ of as-cast PM6:**NiQP** and SVA-treated PM6:**NiQP** are 1.45 and 1.39 eV, respectively. The total *E*_loss_ decomposed into three components: Δ*E*_1_, Δ*E*_2_ and Δ*E*_3_. Δ*E*_1_ is defined as *E*_g_-*qV*_OC_^SQ^, where *qV*_OC_^SQ^ represents the maximum theoretical open-circuit voltage predicted by the Shockley–Queisser (SQ) model, which is nearly identical for both devices. Δ*E*_2_, corresponding to radiative recombination losses below the bandgap, can be expressed as *qV*_OC_^SQ^-*qV*_OC_^Rad^. This loss is influenced by the degree of energetic disorder in the active layer, typically quantified by the Urbach energy (*E*_U_). A lower *E*_U_ indicates reduced energetic disorder, which in turn minimizes radiative recombination losses. The low-energy region of the FTPS-EQE, variation of ln­(EQE) with energy plots (Figure S13), was used to determine the *E*_U_ values for these devices are estimated as *Eu*= (d­(ln *E*QE)/d*E*]^−1^.^40^ The *E*_U_ gives the information about the energetic disorder in the active layer. The *E*_U_ for the device incorporating the SVA-treated active layer is approximately 29.02 meV, lower than that for the as-cast counterpart (30.65 meV). The decrease in *E*_U_ suggests reduced energetic disorder, which may arise from improved molecular packing and lower reorganization energy induced by SVA treatment. Δ*E*_3_ represents the nonradiative energy loss arising from charge carrier recombination within the device and is defined as Δ*E*_3_ = *qV*_OC_^rad^-*qV*_OC_. The Δ*E*_3_ value for the device based on the SVA-treated device is lower than that of the as-cast counterpart, indicating suppressed nonradiative recombination, which contributes to enhanced device performance. Although the OSC incorporating the SVA-treated active layer exhibits reduced *E*_loss_, its *V*_OC_shows a slight decrease. This behavior may arise from increased radiative recombination and SVA-induced shifts in the energy levels, which can partially offset the benefits of reduced energy loss.^28^

### 2.7 Morphological Analysis

Atomic force microscopy (AFM) provides essential insight into the nanoscale morphological organization of donor–acceptor blends, which strongly influences charge separation, charge transport, and the overall power conversion efficiency (PCE) of organic solar cells (OSCs). An appropriate domain size is crucial, as excessively small or large domains can hinder efficient exciton dissociation and charge transport.

To examine the influence of solvent vapor annealing (SVA) on the active-layer morphology, AFM images were recorded, as shown in Figure 6a,b. The root-mean-square roughness (Rq) of the SVA-treated film is approximately 1.65 nm, higher than that of the as-cast film, 1.05 nm. This result suggests that SVA treatment induces a more favorable nanoscale morphology with optimized donor–acceptor domain formation in the PM6:**NiQP** bulk heterojunction active layer. Such morphological optimization facilitates exciton dissociation and charge transport, leading to improved FF, *J*_SC_, and PCE in OSCs based on the SVA-treated PM6:**NiQP** film.^41^ The increased nanoscale roughness is also consistent with reduced energetic disorder in the active layer, as evidenced by the lower Urbach energy (*E*_U_) and reduced energy loss in OSCs incorporating the SVA-treated PM6:**NiQP** BHJ active layer.

**Figure 6:**
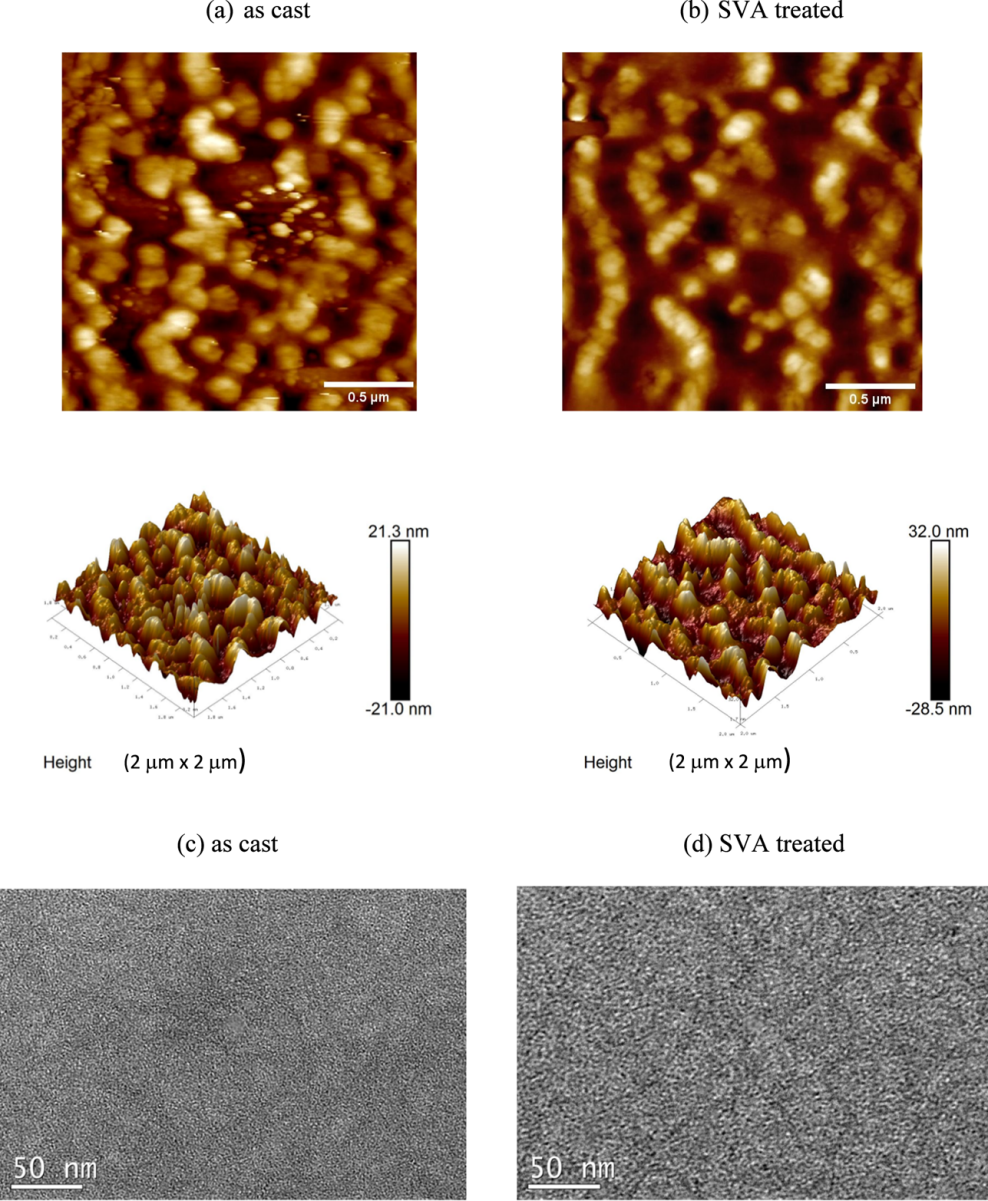
AFM images of (a) as cast and (b) SVA treated PM6:**NiQP** thin films. TEM images of (c) as cast and (d) SVA treated PM6:**NiQP** thin films.

To gain deeper insight into the phase separation behavior of the as-cast and SVA-treated blend films, TEM measurements were performed, and the corresponding images are presented in Figure 6c,d. Compared with the as-cast film, the SVA-treated film exhibits slightly enhanced nanoscale phase separation. Such an optimized morphology can facilitate exciton dissociation and improve charge transport, thereby contributing to the increased short-circuit current density (*J*_SC_) and fill factor (FF) observed in OSCs incorporating the SVA-treated active layer.

## 3 Conclusions

In this work, we have successfully designed and synthesized a novel quinoidal porphyrinoid -based electron acceptor, **NiQP**, and demonstrated its application in OSCs in combination with the donor polymer PM6. The **NiQP** acceptor exhibits a broad absorption profile extending into the near-infrared region and a narrow optical bandgap of approximately 1.44 eV, enabling complementary light harvesting with PM6. The optimized PM6:**NiQP** blend delivered a power conversion efficiency (PCE) of 12.51% after solvent vapor annealing (SVA), representing a significant improvement over the as-cast device (8.47%). This enhancement is primarily attributed to increased short-circuit current density (*J*_SC_) and fill factor (FF), resulting from improved nanoscale morphology, more efficient exciton dissociation, and balanced charge transport.

Comprehensive photophysical and electrical analyses revealed that SVA treatment enhances exciton lifetime and diffusion length, suppresses bimolecular and trap-assisted recombination, and reduces trap density in the active layer. Transient measurements confirmed faster charge extraction and longer carrier lifetimes in SVA-treated devices. Furthermore, energy loss analysis showed that SVA treatment reduces both radiative and nonradiative recombination losses, as evidenced by a lower Urbach energy and smaller Δ*E*_3_ component, indicating diminished energetic disorder and improved molecular packing.

Overall, this study demonstrates that quinoidal porphyrinoids, exemplified by **NiQP**, are promising electron acceptors for high-performance OSCs. The results highlight the potential of molecular engineering combined with postprocessing strategies such as SVA to achieve efficient and stable n-type materials for next-generation organic photovoltaics. Finally, we are working on extending this study to other quinoidal porphyrinoids.

## 4 Experimental Section

### 4.1 Synthesis of Compound NiQP

Under argon atmosphere, at 0 °C, sodium hydride (60% dispersion in mineral oil, 0.24 g, 6 mmol) is added portion wise over a stirred solution of 3-ethyl-2-thioxo-1,3-thiazolidin-4-one (**2**) (0.645 g, 4 mmol) and the resulting mixture is stirred for 30 min at this temperature. This solution is transferred, under argon atmosphere, via cannula, over a stirred solution of 5,15-dibromo-10,20-dimesityl Nickel­(II)^22^ (**1**) (0.3 g, 0.4 mmol), pyridine (1.5 mL), Pd_2_dba_3_ (0.037 g, 0.04 mmol), copper­(I) iodine (0.016 g, 0.08 mmol) and triphenylphosphine (0.032 g, 0.2 mmol) in 150 mL of dry THF. The final reaction mixture is stirred under reflux for 40 h, then the solvent is evaporated, and the crude mixture is filtered through silica gel (Hexane/Toluene 1:1). The solid obtained is solubilized in dichloromethane and treated with 2,3-Dichloro-5,6-dicyano-*p*-benzoquinone (DDQ) (0.454 g, 2 mmol). After 5 min at room temperature, the solvent is evaporated and the crude is purified by column chromatography (Silica gel, Dichloromethane) to obtain a mixture of cis and trans isomers of **NiQP**, as a deep green solid (0.250 g 83% yield) ^1^H NMR (400 MHz, CDCl_3_) δ (ppm): cis isomer:6.97 (s, 2H), 6.93 (s, 1H), 6.90 (s, 1H), 6.84 (d, 2H, *J* = 4.5 Hz), 6.59 (d, 2H, *J* = 4.5 Hz), 6.44 (d, 2H, *J* = 4.5 Hz), 6.36 (d, 2H, *J* = 4.5 Hz), 4.16 (m, 4H), 2.53 (s, 3H), 2.48 (s, 3H), 2.37 (s, 6H), 1.98 (s, 3H), 1.90 (s, 3H), 1.27 (t, 6H, *J* = 7.0 Hz); *trans*-isomer: 6.97 (s, 2H), 6.92 (s, 2H), 6.86 (d, 2H, *J* = 4.6 Hz), 6.55 (d, 2H, *J* = 4.5 Hz), 6.43 (d, 2H, *J* = 4.6 Hz), 6.36 (d, 2H, *J* = 4.5 Hz), 4.16 (m, 4H), 2.50 (s, 6H), 2.36 (s, 6H), 1.95 (s, 6H), 1.27 (t, 6H, *J* = 7.0 Hz). ^13^C NMR (100 MHz, CDCl_3_) δ (ppm): 192.6, 190.6, 190.4, 164.9, 164. 8, 153.2, 152.3, 149.6, 149.0, 146.5, 145.1, 144.5, 139.4, 138.9, 138.6, 138.3, 138.3, 138.2, 138.01, 137.2, 137.1, 137.0, 136.3, 136.2, 132.9, 132.7, 132.3, 132.0, 131.8, 131.0, 130.1, 129.5, 128.1, 128.1, 128.0, 127.9, 127.8, 127.7, 126.3, 125.2, 124.6, 124.22, 120.7, 119.7, 40.1, 21.1, 20.5, 20.5, 20.0, 19.9, 19.8, 12.3. EM (*m*/*z*) (ESI-QTOF) calcd for C_48_H_40_N_6_NiO_6_S_4_: 918.1; found, 918.1. FT-IR (ATR) ν (cm^–1^): 2920, 1647, 1559, 1476, 1441, 1334, 1267, 1068, 1014, 834, 805, 726. UV–vis (CH_2_Cl_2_) λ_max_ (nm) (log ε): 265 (4.43), 306 (4.48), 463 (4.92), 726 (4.72).

## Supplementary Material



## Notes

Experimental details, structural characterization spectra, electrochemical data, theoretical calculations figures, details of the fabrication of OSCs and their characterization (PDF)
