# Supplementary material for: Quinoidal Porphyrinoids as High-Performance Electron Acceptors for Organic Solar Cells: Design, Photophysics, and Device Optimization
Source: ACS Appl Mater Interfaces. 2026 Jul 2;18(27):37777–86. doi: 10.1021/acsami.6c06988 (PMC13383269; doi:10.1021/acsami.6c06988)
Supplement: Supplementary file 1 [file am6c06988_si_001.pdf]

Supporting Information

Quinoidal Porphyrinoids as High-Performance Electron Acceptors for Organic Solar Cells: Design, Photophysics, and Device Optimization

Rubén Caballero,[a] Fernando Langa,[a,] Rahul Singhal,[b] Ganesh D. Sharma,[c,*] Pilar de la Cruz[a,*]*

[a]Instituto de Nanociencia Nanotecnología y Materiales Moleculares (INAMOL). Universidad de Castilla-La Mancha, Toledo 45071, Spain. E-mail: Fernando.Langa@uclm.es and Pilar.Cruz@uclm.es

[b]Department of Physics, Malviya National Institute of Technology, JLN Marg, Jaipur (Rajasthan) 302017, India

[c]Department of Physics and Centre for Material Science and Nano-Electronics, The LNM Institute of Information Technology. Jamdoli, Jaipur (Rajasthan) 302031, India. E-mail: gdsharma@lnmiit.ac.in

I. Materials and Synthesis 3
II. Materials Characterization 3
III. Fabrication of OSCs and characterization....3
IV. Collection of spectra 5
V. HPLC profiles....7
VI. Absorption spectra PM6:NiQP blends 8
VII. Electrochemical Measurements 8
VIII. Computational Studies....9

IX. Photoluminescence spectra..10
X. Photovoltaic properties 10

I. Materials and Synthesis

Reagent and solvents were purchased from commercial sources and were used without further purification. Anhydrous solvents, when indicated, were dried using a Pure-Sov 400 or using standard techniques. Chromatographic purifications were performed using silica gel 60 VWR (particle size 0.040−0.063 mm). Analytical thin-layer chromatography was performed using Merck (TLC) silica gel 60 with F254 as indicator.

II. Materials Characterization

^{1}H-NMR spectra were recorded for solutions in a partially deuterated solvent on a Brüker-Topspin AV 400 instrument. Chemical shifts (δ) values are denoted in ppm. Residual solvent peaks have been used as the internal standard: For ^{1}H-NMR, $CHCl_3$: δ = 7.27 ppm. For ^{13}C-NMR, $CDCl_3$: δ = 77.00. FT-IR spectra of neat samples were recorded in a Jasco FT/IR-6800 FT-IR spectrometer with an ATR accessory. Mass spectra (ESI-QTOF) were recorded on a 3200 QTRAP LC-MS/MS. The main peaks are expressed as m/z. Steady state UV/Vis spectra were recorded from solutions in spectroscopic grade solvents on a Shimadzu UV-VIS-NIR spectrophotometer UV-3600 in quartz cuvettes with a path length of 1 cm. Cyclic and Oster-Young square-wave voltammetries were performed in a µAUTOLAB Type II potentiostat, using 0.1M solution of tetrabutylammonium perchlorate in 1,2-dichlorobenzene:acetonitrile 4:1 as a solvent. Solutions were deoxygenated by bubbling argon through prior to each measurement. Experiments were carried out in a one-compartment cell equipped with a glassy carbon electrode, a platinum wire counter electrode, and an $Ag/AgNO_3$ wire as pseudo-reference electrode. All Potentials were checked against the ferrocene/ferrocenium couple (Fc/Fc^+) after each experiment.

III. Fabrication of OSCs and characterization

The solution-processed organic solar cells were fabricated on the ITO-coated glass substrate with the structure ITO/PESOT:PSS/active layer (PM6:NiQP)/PFN-Br/Ag. The ITO-coated glass substrates were cleaned in detergent, and subsequently ultrasonicated in deionized water, acetone, and isopropyl alcohol and dried in a vacuum oven to remove all traces of residues. The photovoltaic performance optimization process was started with identifying the donor to acceptor ratio (weight percentage, varying from 1:0.4 to 1:1.3), and after that, solvent vapor annealing was applied to maximize the performance of the

OSCs. The conjugated polymer PM6 was used as donor (D), and the total concentration of D:A blend mixture was 16 mg/mL in chloroform. The devices were fabricated by depositing PEDOT:PSS as hole transport layer having thickness of 35-40 nm. The active layer was deposited by spin coating (2500 rpm, 60 s) on the top of PEDOT:PSS layer under ambient conditions. For the solvent vapor annealing (SVA), the optimized (as cast 1:1.2 D/A wt ratio) was exposed to the THF vapors for 40s. A thin layer of PFN was spin-coated on the top of the active layer from the methanol solution. The aluminum (Al) electrode was deposited onto the top of PFN layer via thermal evaporation at a pressure less than 10^{-5} Torr. The current-voltage characteristics of the OSCs were measured under illumination intensity of 100 mW/cm^2 (AM1.5 G) using a solar simulator and a Keithley 2400 source meter unit. The External quantum efficiency (EQE) measurements were performed using Bentham EQE system.

Morphology

The surface morphology of the thin films was characterized using a Bruker ScanAsyst-Air Atomic Force Microscope (AFM) operated in tapping mode with a silicon tip on a nitride cantilever. The measurements were performed using a scan aspect ratio of 1, scan angle of 0°, a scan rate of 1 Hz, and 256 samples per scan line, with a peak force frequency of 2 kHz. The acquired AFM images were analyzed using NanoScope Analysis (64-bit) version 1.80 to evaluate surface morphology and determine root-mean-square (RMS) roughness of the films. Transmission electron microscopy (TEM) was conducted on JEOL JEM 2100 with an acceleration voltage of 200 keV. TEM images were recorded in bright-field mode. Samples were prepared by spin coating the solution of the PM6:RCNiQ active phase over Carbon Film Coated Lacey Carbon Supported Copper Grid, grid size 400 mesh.

IV. Collection of spectra

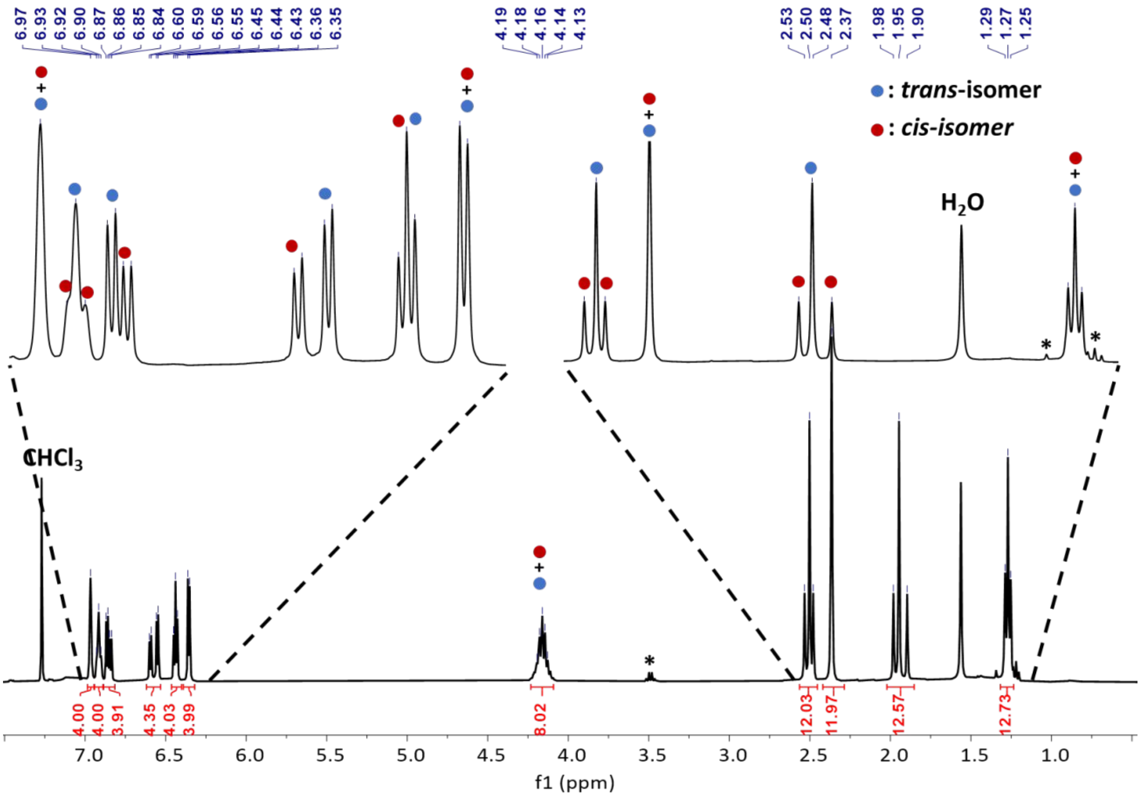

Figure S1. ^{1}H-NMR (400 MHz, $CDCl_3$) spectrum of **NiQP**. (* denotes solvent impurities: methanol and n-pentane)

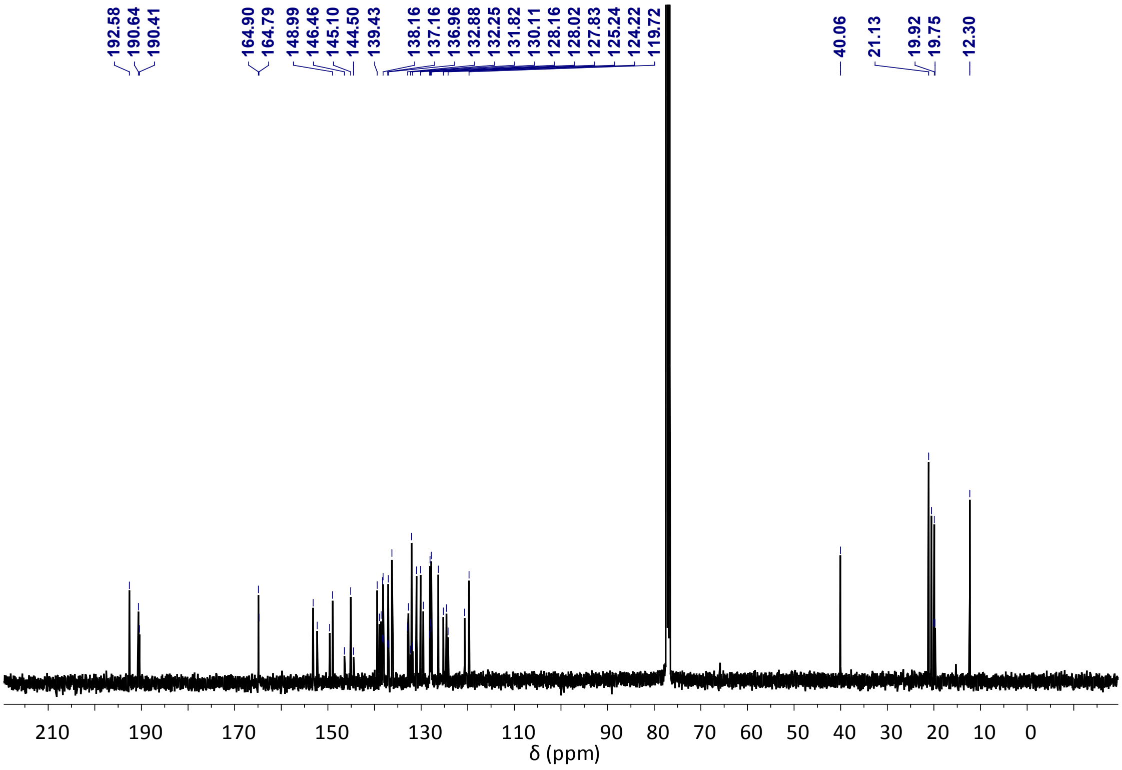

Figure S2. ^{13}C-NMR (100 MHz, $CDCl_3$) spectrum of **NiQP**.

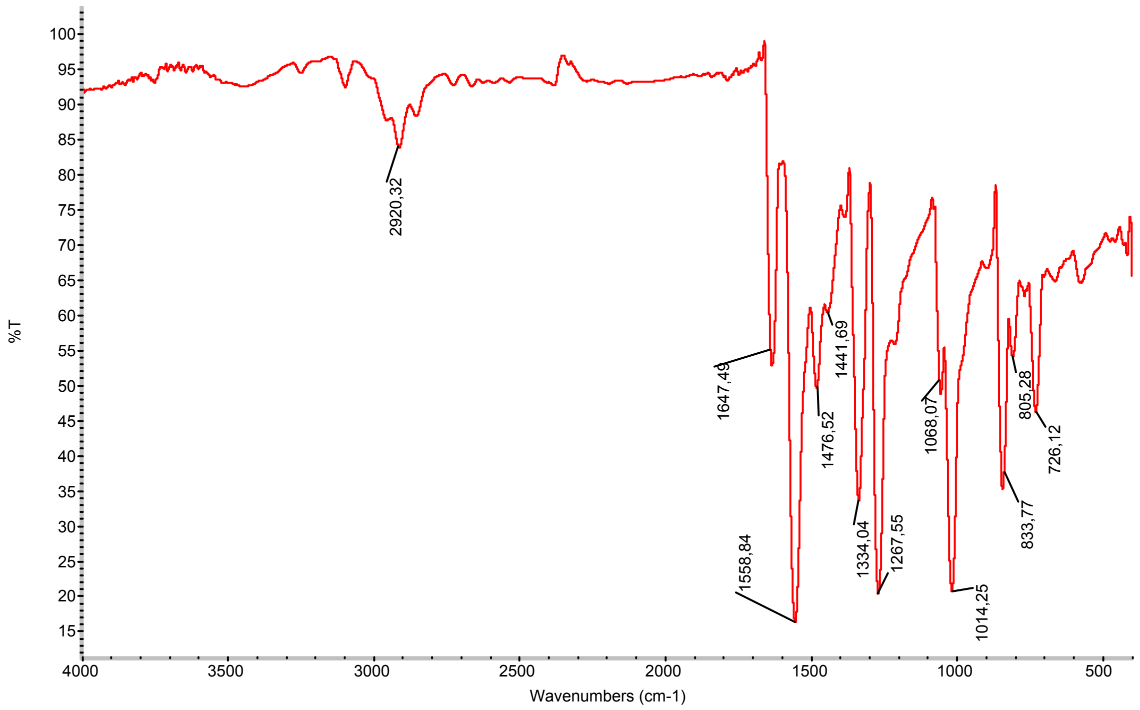

Figure S3. FT-IR (Neat, ATR) spectrum of **NiQP**.

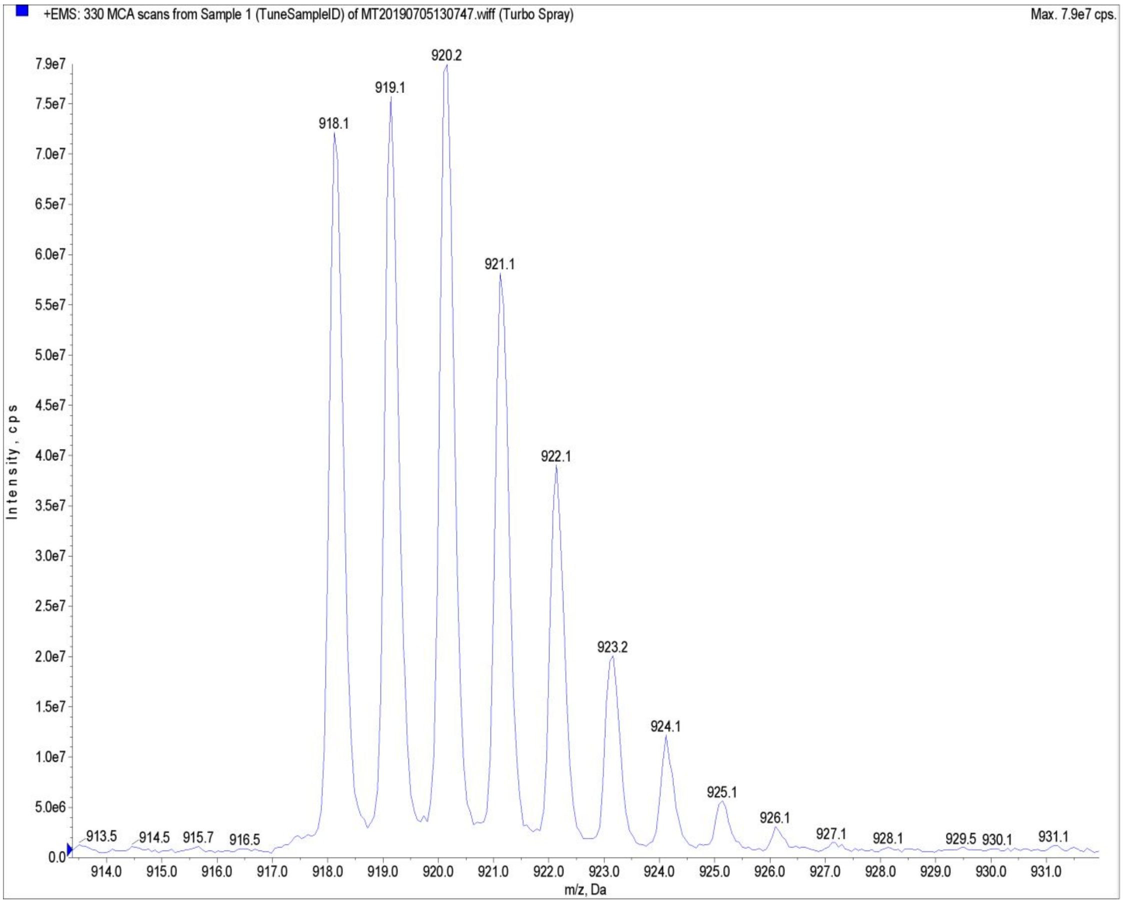

Figure S4. MS (ESI-QTOF) spectrum of **NiQP**.

V. HPLC profiles

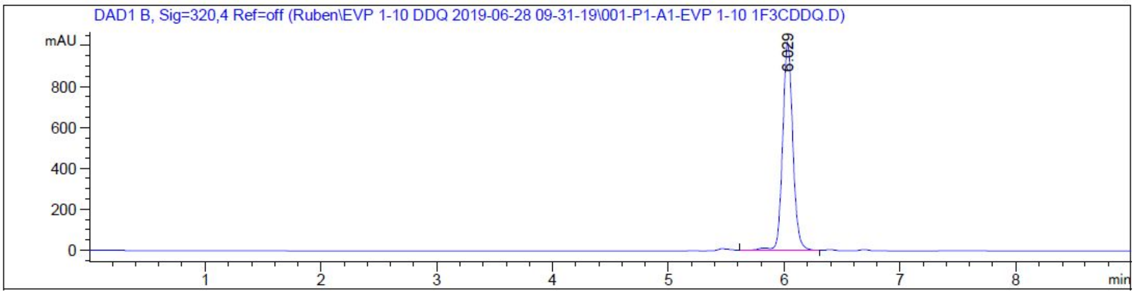

Signal 1: DAD1 B, Sig=320,4 Ref=off

Peak #	RetTime [min]	Type	Width [min]	Area [mAU*s]	Height [mAU]	Area %
1	6.029	VB R	0.0920	6169.04150	1014.82629	100.0000

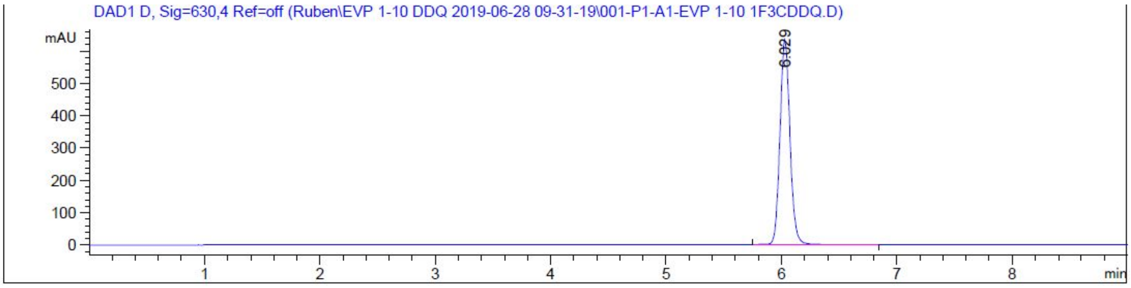

Signal 3: DAD1 D, Sig=630,4 Ref=off

Peak #	RetTime [min]	Type	Width [min]	Area [mAU*s]	Height [mAU]	Area %
1	6.029	BB	0.0920	3809.84546	634.66864	100.0000

Figure S5. HPLC profiles of **NiQP**. Eluent: Toluene, 1mL/min. Column: 2xBuckyprep 4.6IDx250 mm. Up: λ_{obs}= 320 nm Down: λ_{obs}= 630 nm.

VI. Absorption spectra PM6:NiQP blends

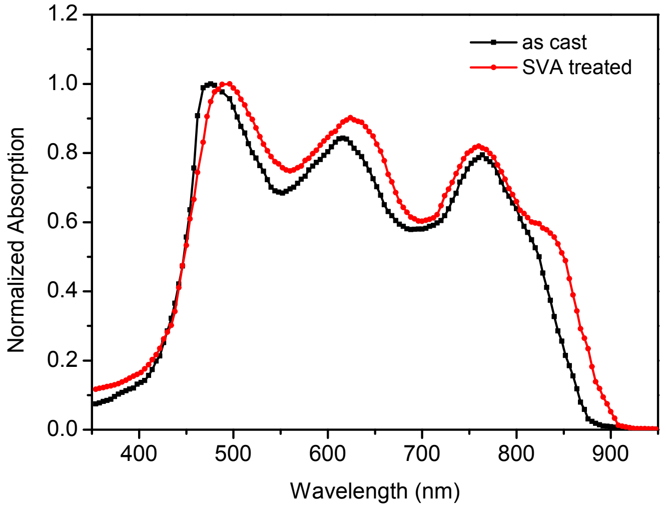

Figure S6. Thin film absorption spectra of as-cast and SVA of PM6:**NiQP** blends.

VII. Electrochemical Measurements

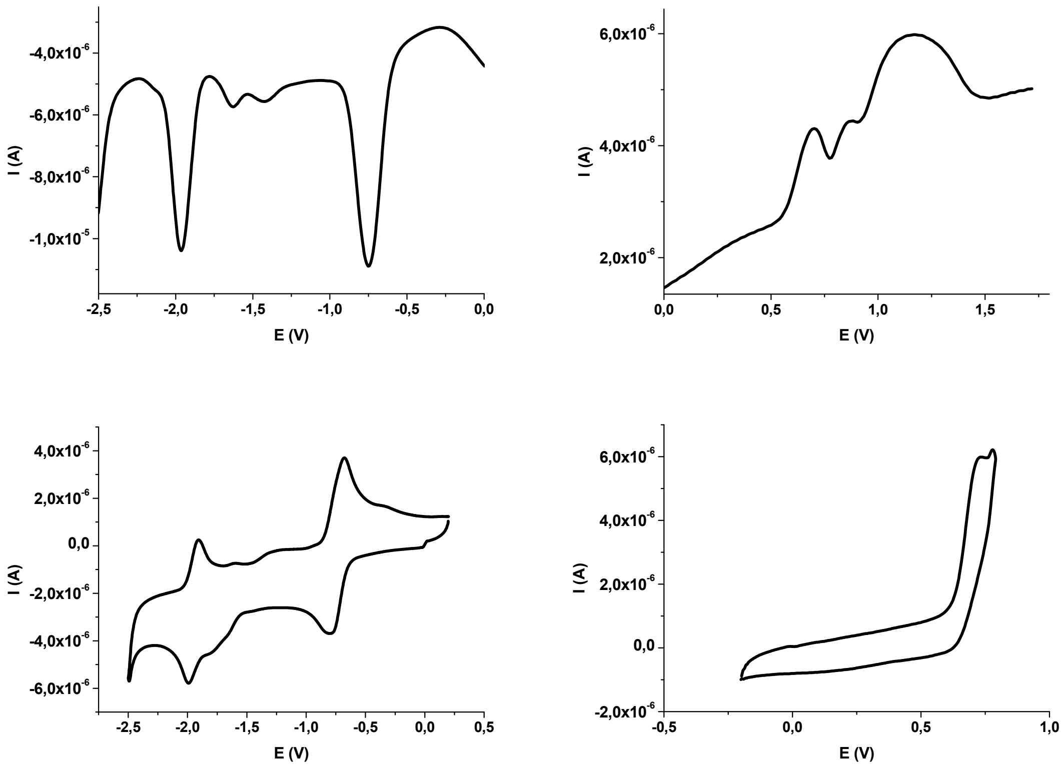

Figure S7. Reduction (left) and oxidation (right) waves of OSWV voltammetry of **NiQP.**

VIII. Computational Studies

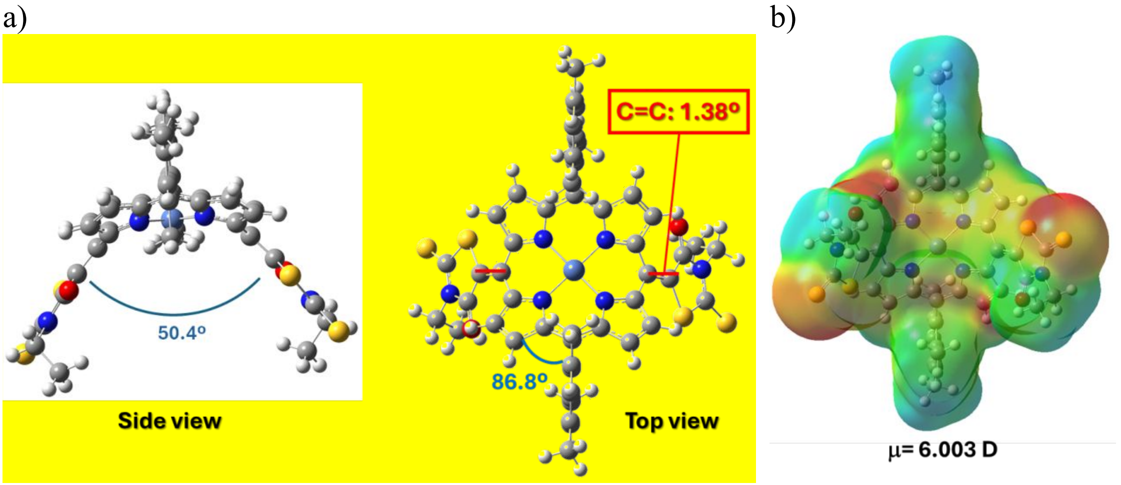

Figure S8. a) Optimized geometry of the quinoidal Ni-porphyrinoid derivative, **NiQP**; b) Electrostatic potential (ESP) surface of **NiQP**, showing the charge-segregation pattern and computed dipole.

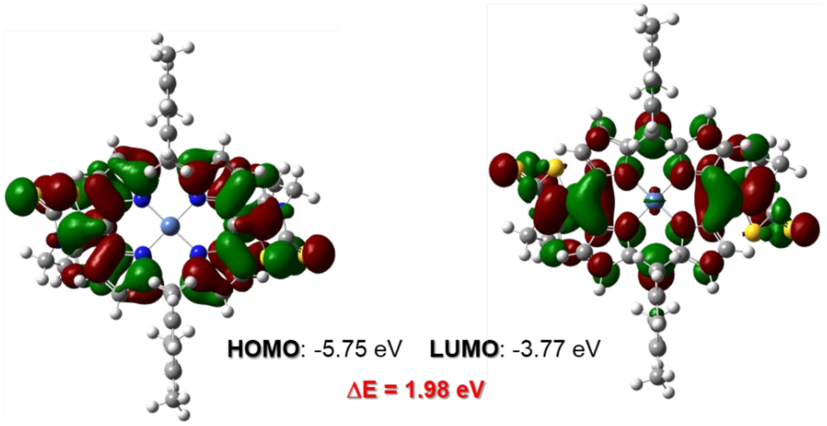

Figure S9. HOMO/LUMO isosurfaces and HOMO/LUMO energy levels of **NiQP**.

IX. Photoluminescence spectra

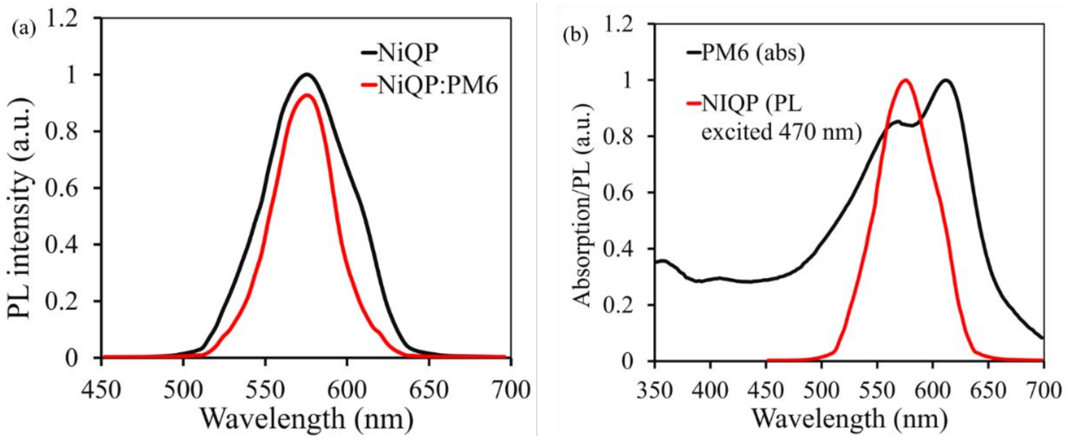

Figure S10. (a) Thin film PL spectra of pristine **NiQP** and its blend with PM6 (**NiQP**:PM6), when excited at 450 nm and (b) PL spectra of **NiQP** (excited at 450 nm) and absorption spectra of PM6.

X. Photovoltaic properties

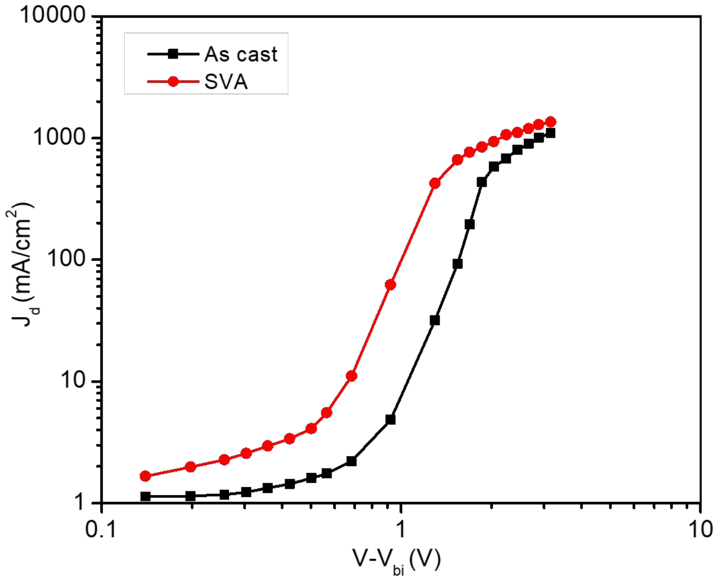

Figure S11. Dark J-V characteristics of ITO/PEDOT:PSS/PM6:**NiQP**/PFN-Br/Al devices based on as cast and SVA treated PM6:**NiQP** active layers.

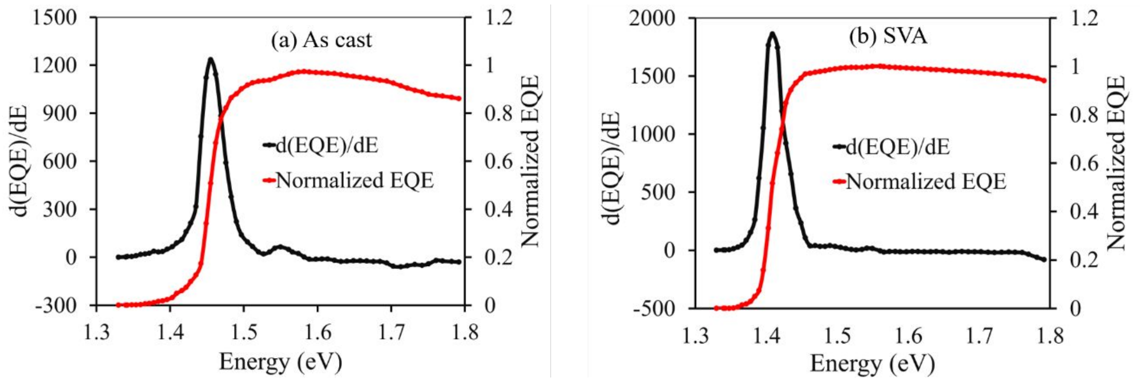

Figure S12. EQE and its first derivative d(EQE)/dE as a function of photon energy E for devices based on (a) as-cast and (b) SVA active layers.

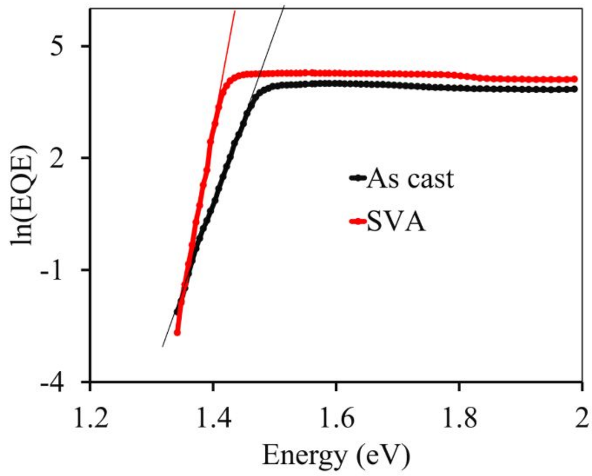

Figure S13. FTPS-EQE (ln EQE) plots for the OSCs based on as-cast and SVA-treated PM6:**NiQP** active layers.

Table S1. Photovoltaic parameters of the binary OSCs based on as cast PM6:**NiQP**.

**Weight Ratio**	**J_{SC} (mA/cm^2)**	**V_{OC} (V)**	**FF**	**PCE (%)**
1:0.4	14.05	0.876	0.534	6.57
1:0.8	15.54	0.881	0.568	7.78
1:1.2	16.45	0.883	0.583	8.47
1:1.4	15.98	0.887	0.572	8.11
